# 

**Published:** 1799-03

**Authors:** 


### MEDICAL AND PHYSICAL JOURNAL;

CONTAINING

THE EARLIEST INFORMATION

ON SUBJECTS OF

*Medicine, Surgery, Pharmacy, Chemistry,*

AND

NATURAL HISTORY,

*AND A CRITICAL ANALYSIS OF ALL NEW BOOKS IN THOSE DEPARTMENTS OF LITERATURE.*

CONDUCTED BY

T. BRADLEY, M. D.

AND

A. F. M. WILLICH, M. D.

174228

S.G.O.L.

Surgeon Genl's Office LIBRARY. 5707 Washington D.C.

—— Ex medicina nihil oportet putare proficisci, nisi quod ad utilitatem corporis spectat, quoniam ejus causâ est instituta.

CICERO, *de Inventione.* Lib. I

VOL. I.

FROM MARCH TO JULY, 1799, INCLUSIVE.

London:

PRINTED FOR R. PHILLIPS,

NO. 71, ST. PAUL'S CHURCH-YARD.

